# A New Concept for Medical Imaging Centered on Cellular Phone Technology

**DOI:** 10.1371/journal.pone.0002075

**Published:** 2008-04-30

**Authors:** Yair Granot, Antoni Ivorra, Boris Rubinsky

**Affiliations:** 1 Biophysics Graduate Group, Department of Bioengineering and Department of Mechanical Engineering, University of California, Berkeley, California, United States of America; 2 Research Center for Bioengineering in the Service of Humanity and Society, School of Computer Science and Engineering, Hebrew University of Jerusalem, Jerusalem, Israel; The University of Queensland, Australia

## Abstract

According to World Health Organization reports, some three quarters of the world population does not have access to medical imaging. In addition, in developing countries over 50% of medical equipment that is available is not being used because it is too sophisticated or in disrepair or because the health personnel are not trained to use it. The goal of this study is to introduce and demonstrate the feasibility of a new concept in medical imaging that is centered on cellular phone technology and which may provide a solution to medical imaging in underserved areas. The new system replaces the conventional stand-alone medical imaging device with a new medical imaging system made of two independent components connected through cellular phone technology. The independent units are: a) a data acquisition device (DAD) at a remote patient site that is simple, with limited controls and no image display capability and b) an advanced image reconstruction and hardware control multiserver unit at a central site. The cellular phone technology transmits unprocessed raw data from the patient site DAD and receives and displays the processed image from the central site. (This is different from conventional telemedicine where the image reconstruction and control is at the patient site and telecommunication is used to transmit processed images from the patient site). The primary goal of this study is to demonstrate that the cellular phone technology can function in the proposed mode. The feasibility of the concept is demonstrated using a new frequency division multiplexing electrical impedance tomography system, which we have developed for dynamic medical imaging, as the medical imaging modality. The system is used to image through a cellular phone a simulation of breast cancer tumors in a medical imaging diagnostic mode and to image minimally invasive tissue ablation with irreversible electroporation in a medical imaging interventional mode.

## Introduction

This study was motivated by several reports on health care and health care technology published by the World Health Organization (WHO) [1]–[5]. According to these reports, the majority of the world population does not have access to health technologies [1], [2]. Affordable and reliable medical imaging technologies are particularly lacking, with grave consequences to the health care of a large part of the world population [1], [3], [4]. It is estimated that some three-quarters of the world's population have no access to medical imaging [1]. Medical imaging devices are often not available in low-income countries with insufficient infrastructure. Furthermore, even when medical imaging devices become available they are often of an inadequate type, non-functional, or handled incorrectly [1]. Around 95% of medical technology in developing countries is imported and over 50% of equipment is not being used, because of lack of maintenance or spare parts, because it is too sophisticated or in disrepair, or because of lack of trained personal [1]. On the other hand, basic medical imaging is taken for granted in industrialized countries, leading to insufficient aid and support being channeled to medical imaging in the developing world [3].

While the WHO reports focus only on the use of medical imaging for diagnostics, in technologically advanced countries medical imaging is being used effectively in minimally invasive or non-invasive imaging monitored and controlled surgical interventions since the early 1980s e.g. [6]–[8]. The field of interventional radiology requires more advanced medical imaging technologies and it is most likely unavailable where diagnostic imaging does not exist.

**Figure 1:**
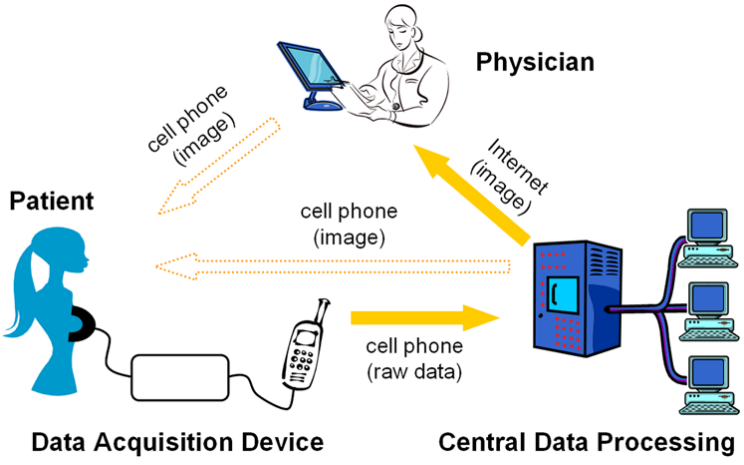
System configuration for the breast cancer tumors patient self-test screening. Outlined arrows indicate optional reporting of results to the patient.

A possible solution to the medical imaging problems detailed in the WHO reports is to develop a different modality for medical imaging. Conventional medical imaging systems are self contained units that combine data acquisition hardware with software processing hardware and imaging display in one device. This causes substantial duplication in expensive components and increases cost. Furthermore, it increases the sensitivity of the devices to user handling and maintenance and places increased demands on users training. Most medical devices have three main components: a) the data acquisition hardware which is in contact with the patient, b) the image display unit and c) the imaging processing hardware. We propose that physically separating these three units could yield a medical imaging technology that is robust, less expensive and can be used by less rigorously trained personnel. The proposed system is illustrated in Figure 1 and is centered on the conventional cellular phone. The system is comprised of a medical imaging data acquisition device (DAD) at the patient site, the cellular phone and a central image reconstruction hardware and software multiserver unit. The conventional cellular phone, which is ubiquitous in many developing countries, can serve as a conduit between the independent medical imaging data acquisition device (DAD) at a remote patient site and the image reconstruction and control hardware and software system, which can simultaneously serve numerous remote patient sites at a central facility. Medical imaging could be done using the DAD at the patient site to acquire the raw data. The cell phone transmits this raw data to a cutting edge central facility that has sophisticated software and hardware for image reconstruction. The data is returned from the central facility to the cellular phone in the form of an image and displayed on its screen. The cell phone also receives and transfers control input to the DAD at the patient site. It should be emphasized that unlike conventional telemedicine where the image reconstruction and control is at the patient site and telecommunication is used to transmit images from the patient site e.g. [9], in our system the cellular phone transmits unprocessed raw data from the medical image DAD at the patient site and receives the processed image.

This design simplifies the apparatus at the patient site and removes the need for advanced imaging training of the personnel at the patient site. It reduces the cost of medical imaging devices in general since the patient site device is only for data acquisition. It also reduces the complexity of operating the hardware at the patient site. The medical imaging display is on a conventional and portable cellular phone and the image is reconstructed at a technologically advanced distant site. This means that there is no need to manipulate the imaging software at the patient site and furthermore, an optimal quality of imaging can be obtained. In addition, because the cellular phone is portable and replaceable its maintenance is minimal. From an economical point of view, the proposed system removes duplications which exist in all the self contained medical imaging devices and should reduce cost. The fact that the image itself is produced in a centralized location and not on the measurement device has additional advantages. Employing a centralized location for image reconstruction would also make technological advances in medical imaging processing continuously available to remote areas of the world. Experts affiliated with the centralized location can analyze the data and the software in the centralized location can be continuously upgraded. Furthermore, control inputs to the remote location (patient site) device as well as advice to the operator at the patient site can be continuously given from the centralized location.

The goal of this study is a first order proof of the feasibility of the medical imaging concept proposed above. We primarily intend to show that the conventional cellular phone can serve the proposed functions. The concept could be developed with various medical imaging modalities, such as ultrasound or even x-ray. It would be most economical with medical imaging systems in which the data acquisition hardware is relatively inexpensive and which requires substantial computation for image reconstruction.

Electrical impedance tomography (EIT) is one of several medical imaging modalities that have attributes compatible with the economical use of the concept [10]. EIT use measurements of currents and voltages from a set of electrodes placed outside the tissue or the body to produce an image of the interior, displayed as a map of the electrical impedance [11]. This can be used, for example, to detect cancer tumors, e.g. [12] or monitor minimally invasive surgical procedures, such as electroporation (the permeabilization of the cell membrane with electrical pulses for genetic engineering, drug delivery, or tissue ablation), e.g. [13]. The data acquisition device (DAD) of EIT is simple and inexpensive. The main components are: a set of electrodes (attached to the object and either inject currents or measure voltages); an AC current source (injects a predefined set of currents to the electrodes); A/D converters (measures the voltages from the electrodes); a simple controller (controls all the other components and the process flow including current injection order, voltage measurements, data collection and organization); and a communication port (communicates with the cell phone, e.g. via Bluetooth or a standard wired link). The DAD does not require a powerful CPU, hard disk or memory space or even a graphical display. On the other hand the EIT image reconstruction software is computationally demanding. The image is reconstructed through a solution of the so-called “inverse problem” i.e. determining impedance distribution inside the object from electrode current and voltage measurements around the object, e.g. [14]. Since the formulation of the problem is inherently ill-posed in the mathematical sense, adequate reconstruction of the data into an image requires elaborate calculations that necessitate powerful signal processors and computer memory [10], [11]. (It should be noted that current breast cancer detection systems already employ as many as 256 electrodes) [15].

The primary goal of this study is to demonstrate that the cellular phone technology can function in the proposed mode. The feasibility of the concept is demonstrated using a new frequency division multiplexing electrical impedance tomography system, which we have developed for dynamic medical imaging, as the medical imaging modality. The system is used to image through a cellular phone a simulation of breast cancer tumors in a medical imaging diagnostic mode and to image minimally invasive tissue ablation with irreversible electroporation in a medical imaging interventional mode.

## Materials and Methods

The key aspect of the new medical imaging concept is the availability of ubiquitous cellular phones and their ability to serve as a conduit of information between remote sites and for imaging display. As a first step in developing this new medical imaging technology the goal of the technical part of this study is limited to a demonstration of the ability of a conventional cellular phone to operate in the proposed mode. Therefore, we did not try to design a simplified and dedicated DAD or an advanced multiprocessors reconstruction system. Instead we used off-the-shelf generic electronic devices and components. Consistent with the mode of operation of the new medical imaging concept, the proof-of-concept system has three components: a) an EIT DAD, b) a cell phone (Palm Treo 700W) (there are a variety of cell phones that could have served equally well) with conventional phone services (Verizon) (other phone services would have served equally well) and c) a processing unit PC (Dell Precision 690, Dual Core Xeon 3.7 GHz, 8 GB SDRAM) completely independent from the DAD.

EIT is based on the measurement of external voltages by means of electrodes placed at specific locations on the body surface. The voltages are induced by different independent currents injected into the body, also through external electrodes. The main role of the DAD is to generate the independent currents and to measure the resulting voltages. The EIT architecture chosen for this study is based on the Frequency-Division Multiplexing EIT (FDM EIT) technique that we have reported previously [16]. In FDM EIT, AC currents at different frequencies are injected simultaneously from all the current injection electrodes and the contribution from each source is isolated after voltage measurements by means of signal processing. Compared with other EIT architectures, the FDM EIT technique is particularly valuable for dynamic imaging.

**Figure 2:**
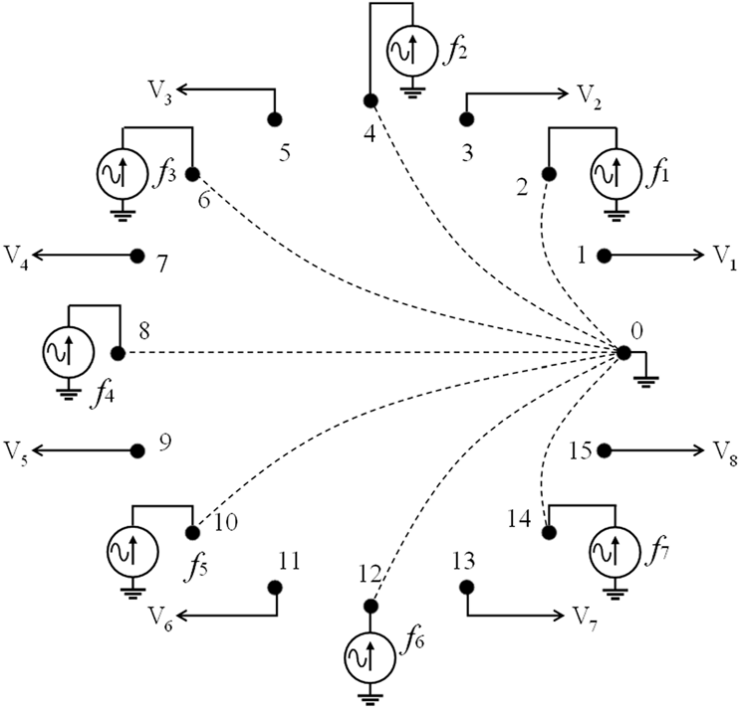
Schematic representation of the Frequency-Division Multiplexing EIT technique that has been employed for the proof-of-concept system (only 16 electrodes out of the actual 32 electrodes are shown for clarity). Seven AC currents (at different frequencies) are injected simultaneously. Signals from voltage electrodes (V1 to V8) are connected to an analogue multiplexer (see Figure 3).

**Figure 3:**
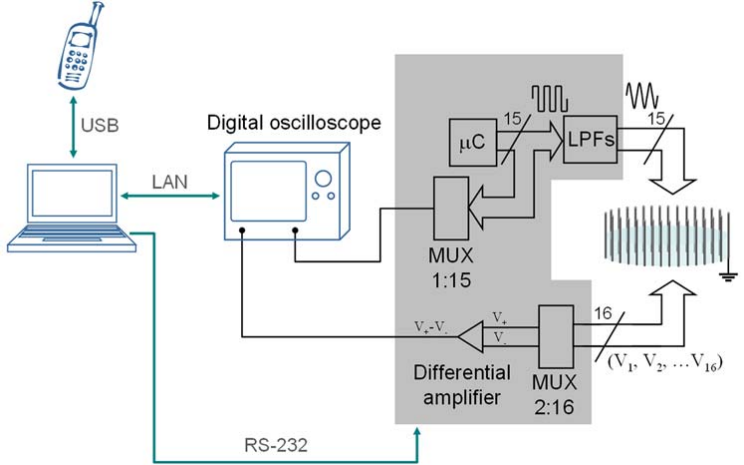
DAD architecture of the proof-of-concept system. The gray shaded area contains the elements that were implemented on a single printed circuit board: a microcontroller (not shown) reads incoming commands from the computer (through the RS-232 connection) and, according to these commands, manages the digital control lines of the analog multiplexers (MUX 1∶15 and MUX 2∶16).

Figure 2 and Figure 3 show the main parts of the DAD implemented for this proof-of-concept study. The system is composed of 32 stainless steel electrodes (length = 20 mm, diameter = 1 mm) around a circular container (diameter = 65 mm). Here 15 electrodes are used as current sources, one as the current sink and the other 16 for voltage measurements. Each current electrode injects an AC current (amplitude = 80 µA) at a different frequency. These frequencies are all in a frequency band (5 kHz to 20 kHz) for which the conductance of physiological solutions or gels is constant.

The injected AC currents are obtained from square signals generated by a set of low cost micro-controllers (PIC16F76 by Microchip Technology, Inc.) that are filtered by second-order low-pass filters (LPFs) with a quality factor (Q) of 4 and centered at the frequency of interest.

A differential amplifier (AD830 by Analog Devices, Inc.) is connected sequentially to different voltage electrode pairs by means of an analogue multiplexer (MUX 2∶16). The signal is then acquired by a digital oscilloscope (LeCroy, WaveRunner 44Xi). The same oscilloscope also records the voltages from the current injectors through another analogue multiplexer (MUX 1∶15). All the recorded signals are acquired by a laptop computer (IBM ThinkPad T43) with a LAN connection to the oscilloscope.

The whole process is performed through custom developed LabVIEW routines (National Instruments Corporation, Austin, TX).

In FDM EIT the voltage measurements are separated according to frequency. The different current patterns that were injected simultaneously are correlated with the voltage measurements. The signal processing routines that extract the voltage data are based on the Fourier transform and are implemented in Matlab (www.mathworks.com). In the last step of processing at the cell phone site, the computer transmits the resulting raw data through the cell phone by means of a USB connection. The format of the raw data is detailed below.

The DAD is connected to the cell phone, via a standard USB cable. The cell phone connects to the data processing computer via a modem by dialing directly to the data processing computer using a Telnet application. The cell phone is used for several tasks – as a display, as a communication link to the processing center, and as a graphical user interface that connects to the DAD and allows the user to initiate a test, or modify settings and other local control functions.

The measurement process begins by powering the DAD and connecting the electrodes. Then the cell phone connects to the DAD via the USB data cable interface. The controller runs the process of injecting current and measuring voltages, collecting the demodulated results into a matrix. There are various ways to generate EIT data [11]. Here we have used 16 electrodes for current injection and 16 for voltage measurement, as shown in Figure 2. A total of 15 electrodes inject a current of to a single sink as explained above, but various other patterns may be used as well. For each current there are 15 independent voltage pair measurements (electrodes 1–3, 3–5, …, 29–31) which were obtained by the FFT as detailed above. Since there are 15 current injections and for each one 15 voltage measurements we have a total of 225 measurements.

The measurements are arranged in a matrix to be transmitted to the processing center. Every measurement is written in a row of the matrix. The columns describe the injected signal's frequency, the injecting electrode number, the positive voltage electrode number, the negative voltage electrode number, the measured voltage amplitude and the phase. For predefined patterns, it is sufficient to report only the last two columns. In our example this matrix is 225×6 and its size is 4 kB. This matrix is uploaded to the cell phone, which dials to the processing center and uploads the matrix though standard data links such as HyperTerminal.

In the data processing computer we have used a Matlab program based on EIDORS [17] to reconstruct the image which was sent back to the cell phone in the form of an ordinary multimedia message using the cell phone service provider's standard web-based interface.

In order to reconstruct the image from the voltage measurements that were sent from the cell phone we solve the Laplace equation over the entire tissue:
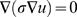
(1)where σ is the electrical conductivity and *u* the electric potential. As explained above, we have injected a set of currents known as a current pattern and from the voltage measurements, which give the boundary conditions of the tissue, we compute the internal conductivity σ. We use the Finite Element Method (FEM) [17] to compute the voltages which result from applying the current pattern and compare them to the measured voltages. When they match we have the correct conductivity.

## Results and Discussion

The feasibility of the cellular phone enabled imaging system was tested by imaging a simulation of breast cancer tumors in a medical imaging diagnostic mode and by imaging image a simulation of minimally invasive tissue ablation with irreversible electroporation in a medical imaging interventional mode.

The diagnostic mode study is also one of the most widely studied uses of EIT and electrical impedance spectroscopy (EIS) [18] and one of the few to become commercially available [19]. The interventional imaging example deals with a new technique for minimally invasive surgery [20] that may become important in rural areas as well, as the requirements for its implementation are relatively modest.

The objective of the tests was to verify that a standard commercial cell phone can be used in two phases of producing the image – connecting the data collection device with the reconstruction center and display of results. We have collected data from an actual EIT system, as described above, and used a cell phone to transmit the measurements. The reconstructed image was sent back to the cellular phone in the form of a standard multimedia message (MMS) and was displayed on the cell phone screen. These tests prove first that the DAD can be separate and independent from the reconstruction process and second that a cell phone can be an effective means of connecting these two parts.

**Figure 4:**
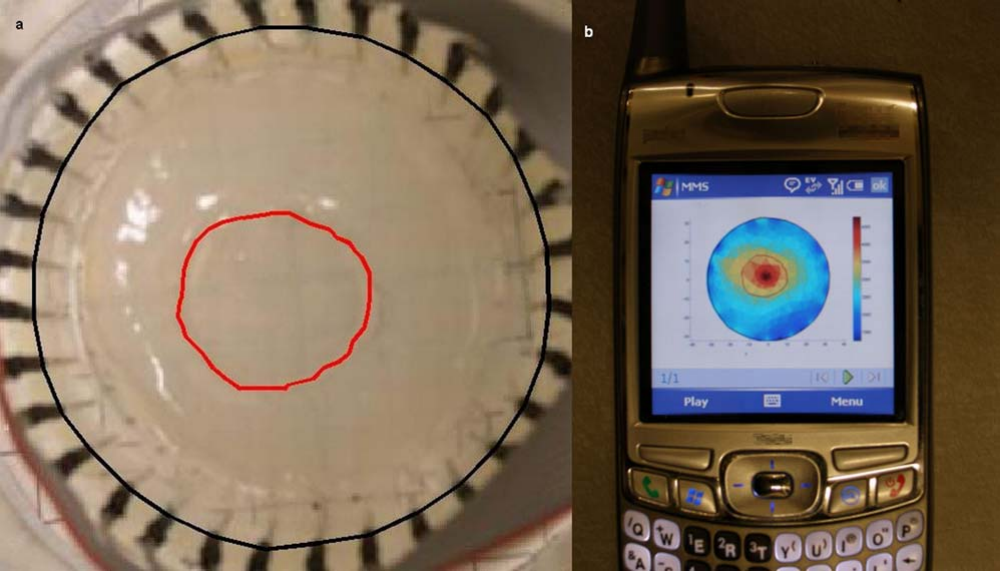
Minimally invasive surgery example. a) The DAD of the system with two types of gel representing an area treated with irreversible electroporation, marked in red, surrounded by normal tissue. b) Reconstructed result as it was displayed on the screen of a commercial cellular phone. Warm colors represent higher conductivity regions that denote an electroporated area.

Figure 4a depicts the EIT DAD with 32 electrodes around a cylinder filled with gel. The device shown here is made from electrically insulating acrylic glass with needles inserted at equal distances around the circumference of the tank. The electrodes are connected to the electrical circuit (not shown here) by means of 2 parallel cables whose wires connect to the electrodes.

The conductivity of the gel is 0.65 mS/cm which is similar to that of a normal liver tissue [21]. We have cut out a cylinder in the central part of the gel and replaced it with another gel with a higher conductivity of 0.93 mS/cm which is similar to the of a liver after irreversible electroporation [21]. The interface between the two regions was not very clear so it is marked by a red line. An additional line in black marks the boundary of the gel where the electrodes are located.

Figure 4b shows the reconstructed image as it was displayed on the cellular phone's screen. The figure shows that we were able to reconstruct an image from the measurements that were sent over the cellular phone and also, that the reconstructed image depicts a higher conductivity region in the location of the inserted gel. To facilitate comparison between the images we have used red and black lines similar to those in Figure 4a. The lines were drawn by first finding the transformation matrix between the boundary of the tissue in the DAD image and the boundary of the circular mesh in the reconstructed image. This translation is necessary since the two images are not the same size and are not centered. We therefore computed the scaling factor and the translation in pixels between the two images, so that the electrodes in Figure 4a would fit the mesh boundary in Figure 4b. Using the same transformation, we converted the contour of the higher conductivity gel that was marked in red in Figure 4a and the result was drawn in Figure 4b, also in red.

**Figure 5:**
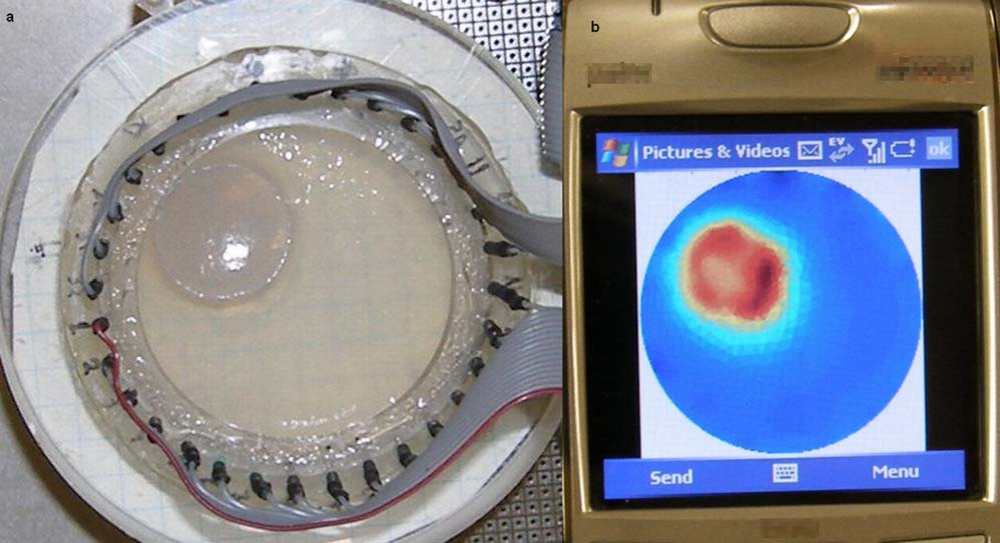
Breast cancer detection example. a) The DAD of the system with two types of gel representing a breast cancer tumor surrounded by normal breast tissue. b) Reconstructed result as it was displayed on the screen of a commercial cellular phone. Warm colors represent higher conductivity regions that are typical of breast cancer lesions.

In Figure 5 we show the results of a breast cancer example. We have simulated a breast cancer tumor (conductivity of 6 mS/cm @ 100 kHz) (upper left side circle) surrounded by normal tissue (0.3 mS/cm @ 100 kHz) [22]. The images are aligned so it is possible to compare the location of the tumor on the mesh in the reconstructed image with that of the picture of the DAD. Alignment was achieved simply by taking the picture from a point of view where the electrode numbers match those of the electrode locations in the mesh.

Another important result of this research is the ease of use of cellular phones in the manner described. This system is easily scalable and could be used in a very similar manner for 3D EIT for example. With the increase in number of electrodes, or the number of current patterns that are used, the size of the measurement matrix increases slightly and in a linear fashion while the requirements from the processing center in terms of memory and processing power increase significantly, usually in a quadratic fashion. This makes the system scalable with only small changes at the DAD, which is typically the hardest place to implement changes in terms of logistics and cost.

The goal of this study was to first and foremost demonstrate that the conventional cellular phone can be used as a central component in the new concept of medical imaging. While in this study we have used the cellular phone as a wireless device with a PC interface there are several options for the use of the cell phone as a communication channel. First is the use as a simple modem. Depending on the cell phone model, many phones on the market today have either a built-in option or a possible add-on to enable them to function as a modem. This usually works with a PC, but a modem interface can be also included in the DAD. The configuration may be complex for this case, depending on the cell phone model and on the DAD hardware that is chosen. The more the DAD resembles a PC interface, the easier the connection will be, but this will also increase the cost and size of the DAD. A second option is to upload the data to the cell phone and to send it using the cell phone's links such as Email, texting / MMS options or Telnet. This depends on the types of service that the cellular provider supports, but at least text messages are a widely available option today, even in the simplest cellular networks. The third option is a customized modem. To be completely independent of the cell phone model, it is possible to implement the modem on the DAD with a suitable speaker that would match an ordinary cell phone microphone. In this case, the cell phone uses the voice channel to transmit an analog signal (much like a fax). This has the disadvantages of possible lost bits due to line quality and other problems that are characteristic of analog communications, but it is probably the best option in terms of cell phone compatibility.

Indeed, an important issue to consider is the requirements for cell phone compatibility. If it is required that the DAD connects to every type of cell phone available the design will have to consider numerous constraints. On the other hand, if the DAD will only be compatible with cell phones that also function as Personal Digital Assistants (PDAs) and run an operating system such as Windows, the design could be very flexible and there are still enough available options on the market for this. Almost every cellular provider, whether it is using GSM, CDMA or other protocols supports a few PDA-like cell phone models that are relatively easy to work with and connect to. An intermediate option, is to limit the compatibility of the DAD to those cells phones that support some minimum features such as USB connection, color display and perhaps a few other things that will be required.

If the cell phone is at least an intermediate option, it may be used as a display/graphical user interface (GUI) for the DAD. Creating the cell phone GUI application depends on the cell phone model and its support of Java or a similar technology. This will make the DAD a little bit simpler since it will not require a built-in display and/or keyboard and the user will not need a PC to use the device (although that is also an option as laptops are widely available). Using the cell phone's keypad, the user can also configure the DAD, run built-in test functions and operate the device.

The phone can be also used for some data processing. This will be limited to the case of using a PDA like model. These cell phones have relatively powerful processing units which may be used to simplify the DAD and move some of the signal processing to the cell phone.

In summary, this study demonstrates the feasibility of using a cellular phone as an integrated part of a medical imaging system in which a robust and independent DAD is connected to the imaging processing site through the cell phone. We believe that this concept has the potential for decreasing the complexity of operating the imaging system at the patient site and make state of the art diagnostic imaging as well as interventional imaging available to people and places that do not have adequate medical imaging now.

## References

[1] WHO report (2003). Essential Health Technologies Strategy 2004–2007. World Health Organization.. http://www.who.int/eht/en/EHT_strategy_2004-2007.pdf.

[2] WHO report, Health Technologies- the backbone of Health Services. World Health Organization.. http://www.who.int/eht/en/Backbone.pdf.

[3] WHO report, Essential Diagnostic Imaging. World Health Organization.. http://www.who.int/eht/en/DiagnosticImaging.pdf.

[4] WHO report, About diagnostic imaging. World Health Organization.. http://www.who.int/diagnostic_imaging/about/en/.

[5] WHO report, Diagnostic imaging. World Health Organization.. http://www.who.int/diagnostic_imaging/en/.

[6] Onik G, Cooper C, Goldenberg HI, Moss AA, Rubinsky B (1984). Ultrasonic Characteristics of Frozen Liver.. Cryobiology.

[7] Gilbert J, Onik G, Haddick W, Rubinsky B (1984). The Use of Ultrasound Imaging for Monitoring Cryosurgery;.

[8] Gilbert J, Onik G, Haddick W, Rubinsky B (1984). The Use of Ultrasonic Imaging for Monitoring Cryosurgery.. IEEE Trans of Biomed Eng.

[9] Kim D-K, Yoo SK, Kim SH (2005). Instant wireless transmission of radiological images using a personal digital assistant phone for emergency teleconsultation.. Journal of Telemedicine and Telecare.

[10] Otten D, Onik G, Rubinsky B (2004). Distributed Network Imaging and Electrical Impedance Tomography of Minimally Invasive Surgery.. Technology in Cancer Treatment and Research.

[11] Bayford RH (2006). Bioimpedance Tomography (Electrical Impedance Tomography).. Annual Review of Biomedical Engineering.

[12] Kao T-J, Isaacson D, Newell JC, Saulnier GJ (2006). A 3D reconstruction algorithm for EIT using a handheld probe for breast cancer detection.. Physiological Measurement.

[13] Davalos RV, Otten DM, Mir LM, Rubinsky B (2004). Electrical impedance tomography for imaging tissue electroporation.. Biomedical Engineering, IEEE Transactions on.

[14] Brown BH (2003). Electrical impedance tomography (EIT): a review.. Journal of Medical Engineering & Technology.

[15] Cherepenin VA, Karpov AY, Korjenevsky AV, Kornienko VN, Kultiasov YS (2002). Three-dimensional EIT imaging of breast tissues: system design and clinical testing.. Medical Imaging, IEEE Transactions on.

[16] Granot Y, Ivorra A, Rubinsky B (2007). Frequency-Division Multiplexing for Electrical Impedance Tomography in Biomedical Applications.. International Journal of Biomedical Imaging.

[17] Vauhkonen M, Lionheart WRB, Heikkinen LM, Vauhkonen PJ, Kaipio JP (2001). A Matlab package for the EIDORS project to reconstruct two-dimensional EIT images.. Physiological Measurement.

[18] Tong In O, Jeehyun L, Jin Keun S, Sung Wan K, Eung Je W (2007). Feasibility of breast cancer lesion detection using a multi-frequency trans-admittance scanner (TAS) with 10 Hz to 500 kHz bandwidth.. Physiological Measurement.

[19] Assenheimer M, Laver-Moskovitz O, Malonek D, Manor D, Nahaliel U (2001). The T-SCANTM technology: electrical impedance as a diagnostic tool for breast cancer detection.. Physiological Measurement.

[20] Rubinsky B, Onik G, Mikus P (2007). Irreversible Electroporation: A New Ablation Modality - Clinical Implications.. Technology in Cancer Research and Treatment.

[21] Ivorra A, Rubinsky B (2006). In vivo electrical impedance measurements during and after electroporation of rat liver.. Bioelectrochemistry.

[22] Surowiec AJ, Stuchly SS, Barr JR, Swarup A (1988). Dielectric properties of breast carcinoma and the surrounding tissues.. Biomedical Engineering, IEEE Transactions on.

